# Targeting *HOX* transcription factors in prostate cancer

**DOI:** 10.1186/1471-2490-14-17

**Published:** 2014-02-05

**Authors:** Richard Morgan, Angie Boxall, Kevin J Harrington, Guy R Simpson, Agnieszka Michael, Hardev S Pandha

**Affiliations:** 1 Faculty of Health and Medical Sciences, University of Surrey, Guildford, UK; 2 Targeted Therapy Team, Chester Beatty Laboratories, The Institute of Cancer Research, London, UK

**Keywords:** Prostate cancer, HXR9, *HOX*, PBX

## Abstract

**Background:**

The *HOX* genes are a family of transcription factors that help to determine cell and tissue identity during early development, and which are also over-expressed in a number of malignancies where they have been shown to promote cell proliferation and survival. The purpose of this study was to evaluate the expression of *HOX* genes in prostate cancer and to establish whether prostate cancer cells are sensitive to killing by HXR9, an inhibitor of *HOX* function.

**Methods:**

*HOX* function was inhibited using the HXR9 peptide. *HOX* gene expression was assessed by RNA extraction from cells or tissues followed by quantitative PCR, and siRNA was used to block the expression of the *HOX* target gene, *cFos. In vivo* modelling involved a mouse flank tumour induced by inoculation with LNCaP cells.

**Results:**

In this study we show that the expression of *HOX* genes in prostate tumours is greatly increased with respect to normal prostate tissue. Targeting the interaction between HOX proteins and their PBX cofactor induces apoptosis in the prostate cancer derived cell lines PC3, DU145 and LNCaP, through a mechanism that involves a rapid increase in the expression of *cFos*, an oncogenic transcription factor. Furthermore, disrupting HOX/PBX binding using the HXR9 antagonist blocks the growth of LNCaP tumours in a xenograft model over an extended period.

**Conclusion:**

Many *HOX* genes are highly over-expressed in prostate cancer, and prostate cancer cells are sensitive to killing by HXR9 both *in vitro* and *in vivo*. The *HOX* genes are therefore a potential therapeutic target in prostate cancer.

## Background

Prostate cancer is the most prevalent male malignancy with just under one million new cases worldwide each year [1]. Treatment pathways for this disease are relatively well defined and include surgery, radiotherapy and/or hormonal therapy. While the majority of patients with early stage disease are cured, 10-15% patient still develop locally recurrent or metastatic disease and have a significantly reduced survival rate [2]. Despite the general adoption of docetaxel chemotherapy agents and novel agents such as abiraterone [3], there is still an urgent need to develop effective new treatments, and therefore it is necessary to explore new target proteins and intracellular signalling pathways.

Recently, considerable interest has been shown in genes that play key roles in defining the identity of cells and tissues in early development and which therefore also have important regulatory roles in cell proliferation and survival. One group of genes that fit into this category are the *HOX* family of transcription factors [4]. HOX proteins are characterised in part by a highly conserved homeodomain that mediates DNA binding, together with a defined set of co-factors that modify their function including members of PBX family [5-7]. The pro-proliferative and anti-apoptotic roles of some *HOX* genes in development make them potential oncogenes, and indeed there are numerous reports of *HOX* overexpression in a range of malignancies, including prostate cancer [4,8-11]. Although definitive oncogenic roles for some *HOX* genes have been described, in general studies on the function of individual *HOX* genes in cancer have been complicated by the high levels of sequence identity and functional redundancy exhibited by most members [12,13]. This functional redundancy in particular has made the results of conventional knock-down studies (using for example siRNA) hard to interpret. As an alternative approach we developed a peptide, HXR9 that acts as a competitive antagonist of the interaction between HOX proteins and their PBX co-factor. This interaction is mediated by a conserved hexapeptide sequence shared by the majority of HOX proteins, and HXR9 can globally repress HOX function through mimicking this peptide [14-22]. In this study we show that prostate tumours have a highly dysregulated pattern of *HOX* expression and that HXR9 induces apoptosis in prostate cancer derived cell lines through a mechanism that involves a rapid increase in expression of the *cFos* gene. Furthermore, HXR9 can block prostate tumour growth *in vivo* for an extended period, suggesting that HXR9 or its derivatives might represent a possible therapeutic option for locally recurrent prostate cancer.

## Methods

### Cell lines and culture

The cell lines used in this study were DU145 (derived from a prostate carcinoma brain metastasis) [23], PC3 (derived from a prostate adenocarcinoma bone metastasis) [24], LNCaP (derived from a prostate carcinoma lymph node metastasis)[25], and WPMY-1 (derived from normal prostate stroma and immortalised with SV40 Large T antigen) [26]. They were obtained from the ATCC through LGC Standards Ltd (UK), and were cultured according to the instructions on the LGC Standards website.

### Synthesis of HXR9 and CXR9 peptides

HXR9 is an 18 amino acid peptide consisting of the previously identified hexapeptide sequence that can bind to PBX and nine C-terminal arginine residues (R9) that facilitate cell entry. The N-terminal and C-terminal amino bonds are in the D-isomer conformation, which has previously been shown to extend the half-life of the peptide to 12 hours in human serum [19]. CXR9 is a control peptide that includes the R9 sequence but lacks a functional hexapeptide sequence due to a single alanine substitution. All peptides were synthesized using conventional column based chemistry and purified to at least 80% (Biosynthesis Inc, USA). The sequences of the peptides are as follows:

HXR9: WYPWMKKHHRRRRRRRRR (2700.06 Da)

CXR9: WYP**A**MKKHHRRRRRRRRR (2604.14 Da)

### Primary prostate tumour RNA

Total RNA from prostate tumours and normal prostate tissue was obtained from OriGene Technologies Ltd, Rockville, USA. Six normal prostate tissue samples (median age of donor 56 years, range 52–71 years), and 17 prostate tumour samples (median age of donor 60 years, range 48–73 years) were included in the analysis. Of the prostate tumour samples, 5 were Gleason grade 6, 8 were Gleason grade 7, 1 was Gleason grade 8, and 3 were Gleason grade 9. Reverse transcription and QPCR were performed as described below.

### RNA purification and reverse transcription

Total RNA was isolated from cells using the RNeasy Plus Mini Kit (Qiagen) by following the manufacturer’s protocol. The RNA was denatured by heating to 65ºC for 5 minutes. cDNA was synthesized from RNA using the Cloned AMV First Strand Synthesis Kit (Invitrogen) according to the manufacturer’s instructions.

### Quantitative PCR

Quantitative PCR was done using the Stratagene MX3005P real-time PCR machine and the Brilliant SYBR Green QPCR Master Mix (Stratagene). Oligonucleotide primers were designed to facilitate the unique amplification of *β-actin*, *c-Fos*, and each *HOX* gene. The expression of each gene was calculated using the ^ΔΔ^Ct method.

### Mice and ***in vivo*** trial

All animal experiments were conducted in accordance with the United Kingdom Co-ordinating Committee on Cancer Research (UKCCCR) guidelines for the Welfare of Animals in Experimental Neoplasia [27]. The experimental protocol was approved by the University of Surrey Animal Welfare Ethical Review Board, and by the UK Home Office (licence number 70/7347).

Athymic nude mice were kept in positive pressure isolators in 12 hour light/dark cycles and food and water were available *ad libitum*. Mice were inoculated subcutaneously with a suspension of 2.5 × 10^6^ LNCaP cells in culture media (100 μl). Once tumours reached volumes of approximately 100 mm^3^, mice received an initial, single intratumoural dose of 100 mg/Kg CXR9 or HXR9 dissolved in 0.1 ml PBS, with subsequent dosing when or if the tumour reoccurred. The HXR9 and CXR9 treatment groups contained 9 and 8 mice, respectively. The mice were monitored carefully for signs of distress, including behavioural changes and weight loss.

## Results

### Multiple *HOX* genes are dysregulated in prostate tumours and cell lines

**Figure 1:**
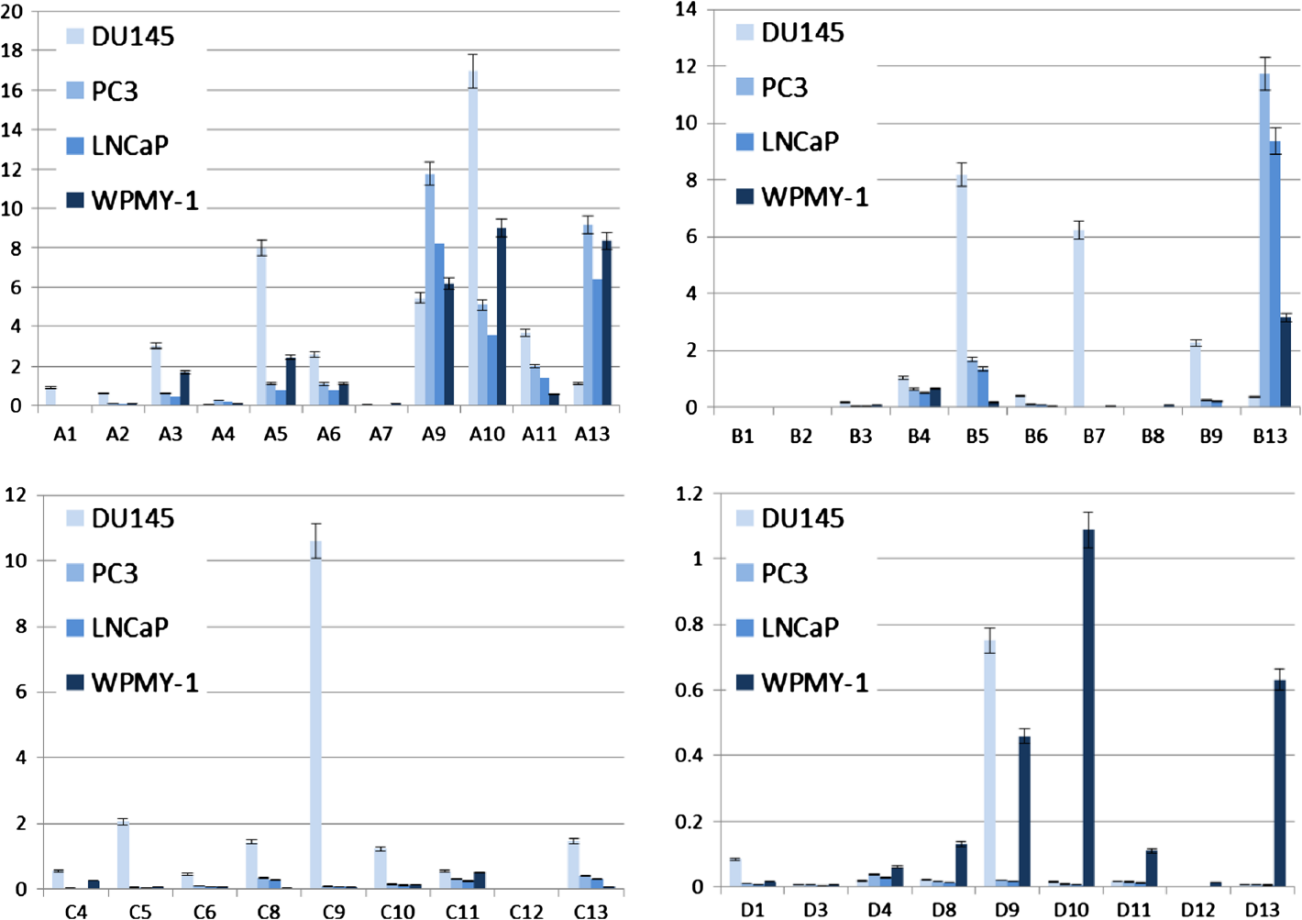
***HOX *gene expression in prostate cancer derived cell lines and in WPMY-1, which is derived from normal prostate cells.** The expression of each gene was determined by semi-quantitative PCR and is shown relative to the house keeping gene *GAPDH* (x10000). The values shown are the mean of three independent experiments and the error bars represent the standard error of the mean (SEM).

Previous studies have revealed that *HOX* genes are generally dysregulated in malignant tissue compared to normal adult cells, and we investigated whether this is also the case in prostate cancer. In order to do this we obtained RNA from three cell lines derived from prostate cancer; DU145 [23], PC3 [24] and LNCaP [25], and a cell line derived from non-malignant prostate stromal cells, WPMY-1 [26]. QPCR analysis of expression levels of all 39 *HOX* genes shows that the tumour derived cell lines have significantly different patterns of *HOX* expression compared to WMPY-1. In particular, the cancer-derived lines all show higher expression of the *HOXC* genes and of *HOXB5* and *HOXB7*, whilst WMPY-1 expresses *HOX* genes closer to the 5′ (posterior) end of each *HOX* cluster (Figure 1).

### The HOX/PBX antagonist HXR9 is cytotoxic to prostate cancer derived cell lines

**Figure 2:**
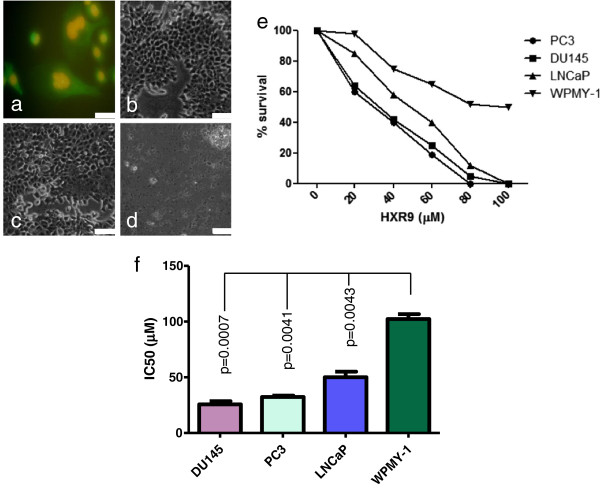
**Prostate cancer-derived cell lines are sensitive to killing by the *HOX*/PBX antagonist HXR9. (a)** HXR9 enters the cytoplasm and nuclei of PC3 cells *in vitro*. PC3 cells were incubated with 5 μM FITC labelled HXR9 (green) for two hours and then stained with Hoechst S769121 (a fluorescent dye staining nuclei yellow). Scale bar: 20 μm **(b-d)** Light micrographs of PC3 cells either untreated **(b)** or incubated with 60 μM CXR9 **(c)** or HXR9 **(d)** for two hours. Scale bar: 100 μm **(e)** Survival curves for PC3, DU145, LNCaP and WPMY-1 cells treated with HXR9. **(f)** IC50 values for HXR9 treatment. The negative control peptide CXR9 was not toxic at any of the concentrations tested for any of the cell lines (i.e. the IC50 > 100 μM). Error bars represent the SEM (n = 3), the p values are with respect to WPMY-1.

Given the elevated expression of *HOX* genes in both primary prostate tumours and cell lines, we assessed whether the prostate cancer-derived cell lines LNCaP, DU145 and PC3 were sensitive to killing by the HOX/PBX antagonist HXR9. HXR9 is an 18 amino acid peptide that can enter cells via endocytosis mediated by a polyarginine sequence. A fluorescently labelled version of this peptide was taken up by all of the cell lines tested (Figure 2a), and could be detected in both the cytoplasm and the nucleus. As a negative control, we used a second peptide, CXR9, which is identical to HXR9 with the exception of a single alanine substitution in the hexapeptide sequence. PC3 cells treated with 60 μM CXR9 for two hours do not exhibit any apparent cytotoxicity compared to untreated cells (Figure 2b, c), whilst the same concentration of HXR9 results in extensive cell death (Figure 2d).

The cytotoxicity of HXR9 and CXR9 was determined for all three cancer-derived lines and the non-malignant line WPMY-1 using an MTS assay. This revealed that all three of the lines were sensitive to killing by HXR9, whilst WPMY-1 cells were significantly less sensitive (Figure 2e, f).

### HXR9 induces apoptosis in prostate cancer derived cell lines

**Figure 3:**
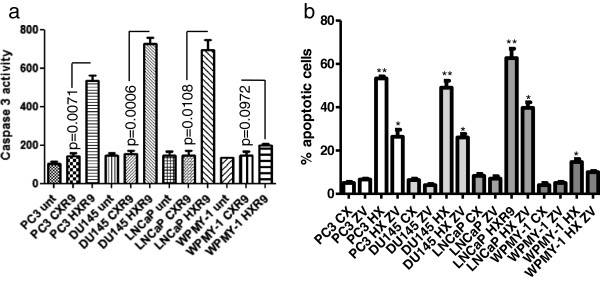
**HXR9 induces apoptosis in prostate cancer cells. (a)** Caspase 3 activity in PC3, DU145, LNCaP and WPMY-1 cells treated with 60 μM HXR9 or CXR9 for two hours. ‘Unt’ - untreated cells; Y axis units: relative fluorescence **(b)** PC3, DU145, LNCaP and WPMY-1 cells were treated with 60 μM HXR9 (HX) or CXR9 (CX) for two hours and cells were assessed for apoptosis through Annexin/7AAD staining. The % of cells in apoptosis is shown. Cells were also treated with the Caspase inhibitor ZVAD (ZV) alone or in combination with HXR9 (HX ZV). Error bars show the SEM. *p < 0.05, **p < 0.01 with respect to untreated cells.

To further understand the mechanism of HXR9-induced cell death, we studied the activity of caspase 3 in HXR9-treated cells. Caspase 3 is a key component of both the intrinsic and extrinsic apoptotic pathway, and can cleave a group of proteins involved in cell survival and proliferation. All of the prostate cancer derived cell lines showed a significant increase in caspase 3 activity when treated with 60 μM HXR9 for two hours (3.7 fold for PC3 cells and 4.8 fold for both DU145 and LNCaP cells), whilst WPMY-1 cells do not (1.4 fold increase, p = 0.0972). Treatment with CXR9 did not change caspase 3 activity in any of the cell lines (Figure 3a).

To further explore whether HXR9 induces cell death primarily through apoptosis, we also used a FACS based analysis for changes in the cell membrane that are characteristic of process and which can be detected by fluorescently labelled Anexxin. The assay also utilises a fluorescent DNA label (7AAD) to measure the membrane integrity of cells, thus allowing cells to be divided into those undergoing early or late stage apoptosis depending on the relative binding of the two labels (Figure 3b). All of the cell lines tested had significantly increased levels of apoptosis after a two hour treatment with 60 μM HXR9, compared to CXR9 treated cells. Apoptosis was considerably higher in the prostate cancer derived cell lines PC3, DU145 and LNCaP (10.6, 8.2 and 7.9 fold, respectively) than in WPMY-1 (3.8 fold).

To provide further confirmation that cells were undergoing apoptosis, HXR9 and CXR9 treated cells were also treated with 50 μM Z-VAD, a caspase inhibitor that blocks the apoptotic cascade. This caused a significant reduction in the proportion of cells undergoing apoptosis (Figure 3b), with the exception of WPMY-1 cells.

### HXR9 induced cell death is mediated by cFos

**Figure 4:**
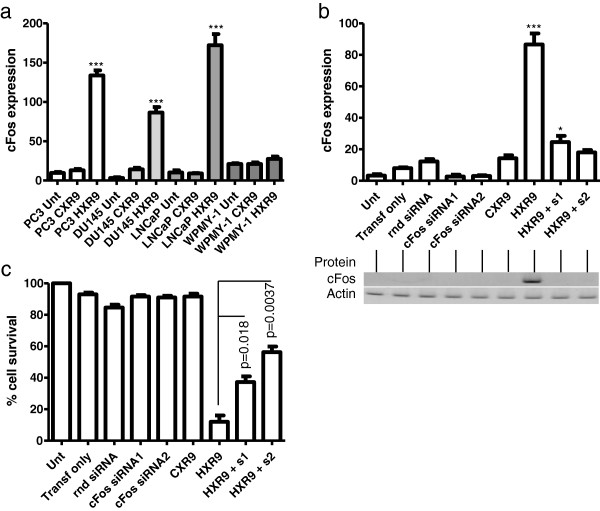
**HXR9 induced apoptosis is mediated by *cFos*. (a)** QPCR analysis of *cFos* expression in response to treatment of PC3, DU145, LNCaP and WPMY-1 cells with 60 μM HXR9 or CXR9 for two hours. ‘Unt’ – untreated cells; *cFos* expression is shown as a ratio with the *β-actin* gene (×10,000). **(b)** Knock down of *cFos* expression using siRNA. DU145 cells were incubated with transfection reagent alone (‘Transf only’), or were transfected with a control siRNA with a random sequence (‘rnd siRNA’), or with one of two siRNA targeting *cFos* (‘cFos siRNA 1’ and ‘cFos siRNA 2’). Cells were also either treated with 60 μM HXR9 or CXR9, or with HXR9 in combination with one of the transfected *cFos* specific siRNAs (‘HXR9 + s1’ or ‘HXR9 + s2’). The expression of *cFos* in treated cells was determined either at the RNA level using QPCR (upper section) or at the protein level using western blotting (lower section). **(c)** The % cell survival for each of the treatments described in **(b)**. Knock down of *cFos* in HXR9 treated cells results in a statistically significant decrease in cell death. Error bars show the SEM. *p < 0.05, ***p < 0.001 with respect to untreated cells.

Previous studies have suggested that HXR9-induced apoptosis might be mediated by the elevated expression of the *cFos* gene [19,21]. To further explore this, and determine whether it is true for prostate cancer derived cells, we used QPCR to assess the expression level of *cFos* in HXR9 and CXR9 treated cells. A two hour treatment with 60 μM HXR9 caused a 6.2, 10.3 and 19.1 fold increase in *cFos* levels in DU145, PC3 and LNCaP cells, respectively (Figure 4a). In contrast, no significant increase was observed in WPMY-1 cells.

In order to establish whether increased *cFos* levels were directly responsible for inducing cell death, we used a siRNA knock down strategy to reduce *cFos* expression in HXR9 treated cells. DU145 cells were transfected with a random control siRNA (rnd siRNA), or one of two different siRNAs designed against the *cFos* sequence (siRNA1/2). Pre-treatment of DU145 cells with either of the *cFos* siRNAs was sufficient to block the increase in *cFos* expression upon subsequent treatment with HXR9, both at the mRNA and protein level (Figure 4b). This also resulted in a significant increase in cell survival (from 12% in cells treated with HXR9 only, to 37% and 56% in cells pre-treated with *cFos* siRNA1 and *cFos* siRNA2, respectively; Figure 4c).

### HXR9 blocks the growth of LNCaP tumours *in vivo*

**Figure 5:**
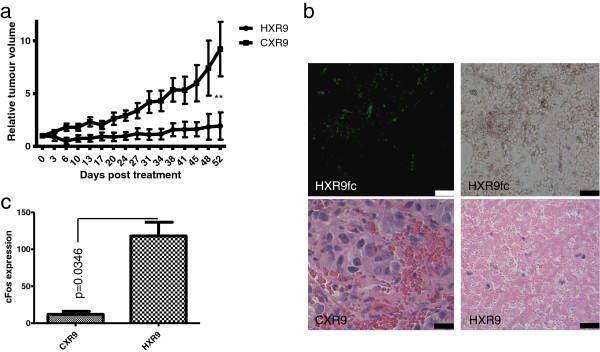
**HXR9 retards LNCaP tumour growth *in vivo*. (a)** Growth curve for LNCaP tumours treated intratumorally with a single dose of HXR9 (9 mice) or CXR9 (8 mice) when the tumour volume reached 100 mm^3^, followed by further doses if the tumour recurred. Error bars show the SEM. “Relative tumour volume” refers to the fold change in tumour size from the time of the first injection. **(b)** Top panels, sections through LNCaP tumours from mice treated with low dose (1 mg/Kg) FITC-HXR9. Top left, fluorescent view showing HXR9-FITC distribution (green). Top right, the same section under light microscopy. Scale bar: 100 μm. Bottom panels, sections through LNCaP tumours from mice treated with 100 mg/Kg HXR9 or CXR9. The CXR9 treated section shows highly undifferentiated tumour cells, whilst the HXR9 section shows the remains of dead tumour cells. Scale bar: 20 μm. **(c)** Expression of *cFos* in tumours treated with HXR9 or CXR9 2 hours prior to their excision, shown as a ratio between *cFos* and GAPDH transcripts detected by QPCR (x10,000).

The sensitivity of prostate cancer derived lines to killing by HXR9 *in vitro* prompted us to test whether this sensitivity was also apparent *in vivo*. We initiated flank tumours in nude mice using a subcutaneous injection of LNCaP cells, which have been used in numerous studies as a murine model of prostate cancer. Tumours were injected directly with HXR9 or CXR9 once the mean tumour volume had reached 100 mm^3^. After 52 days the CXR9 injected tumours had, on average, increased in size 8 fold, whilst the average increase in HXR9 tumours was 1.5 fold (Figure 5a). Histological analysis of tumours revealed that whilst CXR9 treated tumours were composed principally of live, highly undifferentiated cells, those injected with HXR9 contained relatively few cells and were composed to a large extent of cellular debris (Figure 5b). Treating mice with a low dose of fluorescently labelled HXR9 revealed a widespread take up of the peptide by the tumour cells (Figure 5b). QPCR analysis of RNA extracted from tumours revealed that HXR9 induces an up regulation of *cFos* in a similar manner to that seen *in vitro*, suggesting that a similar mechanism of cell death may occur (Figure 5c).

### *HOX* genes are globally overexpressed in primary prostate tumours

**Figure 6:**
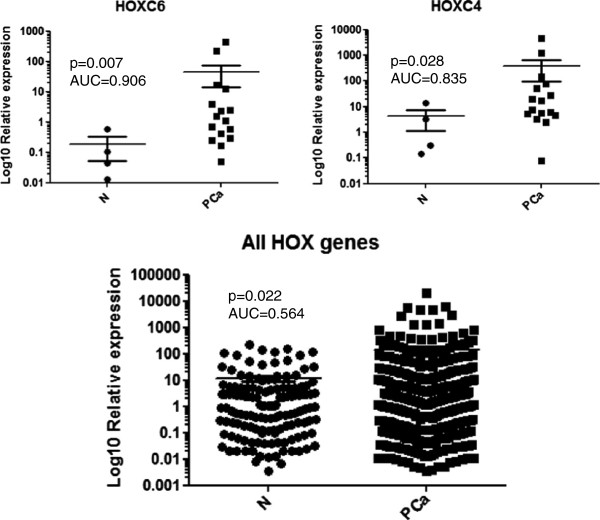
***HOX *gene expression in normal prostate tissue and prostate tumours.** The expression of each gene was determined by semi-quantitative PCR and is shown relative to the house keeping gene *GAPDH* (x10000). The horizontal bar represents the mean and the error bars represent the SEM. AUC, area under the curve from a receiver-operator characteristics analysis.

As previous studies on *HOX* gene expression in prostate cancer have focused only on single or small groups of genes, we undertook an analysis of all 39 *HOX* genes in prostate tumours and normal prostate tissue. This revealed a considerable over expression of many *HOX* family members, albeit in a heterogeneous manner with different *HOX* genes being overexpressed in different tumours. Only *HOXC4* and *HOXC6* showed consistently higher expression in the tumour compared to the normal prostate, with increases of 101 and 251 fold, respectively (Figure 6). Taking all of the *HOX* genes together also revealed a significantly higher global *HOX* expression in tumours (11.9 fold, Figure 6).

## Discussion

In this study we have shown that *HOX* genes are highly deregulated in prostate tumours and in prostate cancer derived cell lines, which concurs with the findings of a number of previous studies [9-11]. It reveals that there is a very high level of deregulation with the majority of *HOX* genes being highly expressed in tumours but not in normal prostate tissue. This global increase in *HOX* expression makes it difficult to study those aspects of *HOX* function that are redundant throughout this highly conserved group. Here we have used HXR9, an inhibitor of the interaction between HOX proteins and their common cofactor, PBX, to target a large subset of HOX proteins (i.e. members of paralogue groups 1-9) [19]. HXR9 causes apoptosis in all three of the prostate derived cells line studied, but only to a far lesser degree in a non-malignant cell line derived from prostate stroma (WPMY-1).

Disruption of HOX/PBX regulated transcription would be expected to cause changes in the expression of numerous target genes, and indeed previous studies have shown this to be the case. However only one of these targets – *cFos* – has been shown to be directly relevant to the induction of apoptosis by HXR9 [19]. It was previously shown that *cFos* up-regulation mediated the HXR9-induced apoptosis in melanoma B16F10 cells, and here we show that a similar mechanism exists in the prostate cancer-derived cell lines DU145, PC3 and LNCaP, as siRNA knock-down of *cFos* can partially rescue each of these cell lines from HXR9 –induced cell death. Although *cFos* is classically considered to be an oncogene, there are now a number of reports of it acting as a pro-apoptotic gene [19,28-31]. Our observation that HXR9 results in a rapid and very large increase in *cFos* expression indicates that the HOX/PBX dimer acts as a repressor of this gene. Whilst this could be a direct result of HOX/PBX binding to its regulatory sequences, a recent study showed that it could also be due to the increased transcription of the oncogenic microRNAs miR221 and mir222, which in turn repress *cFos* expression [16].

The prevalence of *HOX* over expression in prostate cancer combined with the novel therapeutic mechanism exploited by HXR9 suggest that it could be a therapeutic approach where there is small volume, well defined disease. Local delivery of HXR9 into a range of tumours in mice has not resulted in a local inflammatory response [18,20,21]. Therefore delivery of HXR9 directly into the restricted confines of a primary or locally recurrent prostate cancer is feasible, and would not be limited due to the risk of prostatitis. Indeed a number of studies have evaluated intraprostatic gene therapy and oncolytic viral therapy and have reported no dose-limiting toxicity. These approaches utilised current imaging technology to achieve the precise delivery of reagents to small volume, well defined disease [32-34]. The application of HXR9 may be as a primary focal therapy, or where standard treatments approaches have failed, for example in cases of local recurrence following radical radiotherapy. The latter group of men currently receive ablative therapy which has low efficacy and is associated with significant toxicities [35]. In contrast to current treatments such as cryotherapy, the lack of inflammatory response associated with HXR9 treatment would potentially allow multiple, sequential intratumoral delivery.

## Conclusions

The *HOX* genes are highly dysregulated, and generally over-expressed in prostate cancer. Targeting the interaction between HOX proteins and their PBX co-factor is a potential therapeutic strategy in this malignancy.

## Competing interests

The authors declare that they have no competing interests.

## Authors’ contributions

RM designed the study and wrote the manuscript. AB performed the cell viability assays and gene expression analysis. KH critically reviewed the manuscript and helped with experimental design. GS performed the *in vivo* experiments. AM assisted in the primary tissue collection and the analysis of gene expression in these samples. HP helped to design the study and to write the manuscript. All authors read and approved the final manuscript.

## Pre-publication history

The pre-publication history for this paper can be accessed here:

http://www.biomedcentral.com/1471-2490/14/17/prepub

## References

[B1] Ferlay J Shin HR Bray F Forman D Mathers C Parkin DM Estimates of worldwide burden of cancer in 2008: GLOBOCAN 2008 Int J Cancer 2010 127 2893 2917 21351269 10.1002/ijc.25516

[B2] DiBlasio CJ Malcolm JB Hammett J Wan JY Aleman MA Patterson AL Wake RW Derweesh IH Survival outcomes in men receiving androgen-deprivation therapy as primary or salvage treatment for localized or advanced prostate cancer: 20-year single-centre experience BJU Int 2009 104 1208 1214 19388987 10.1111/j.1464-410X.2009.08593.x

[B3] Mukherji D Eichholz A De Bono JS Management of metastatic castration-resistant prostate cancer: recent advances Drugs 2012 72 1011 1028 22621691 10.2165/11633360-000000000-00000

[B4] Shah N Sukumar S The *HOX* genes and their roles in oncogenesis Nat Rev Cancer 2010 10 361 371 20357775 10.1038/nrc2826

[B5] Chang CP Brocchieri L Shen WF Largman C Cleary ML Pbx modulation of *HOX* homeodomain amino-terminal arms establishes different DNA-binding specificities across the *HOX* locus Mol Cell Biol 1996 16 1734 1745 8657149 10.1128/mcb.16.4.1734 PMC231160

[B6] Knoepfler PS Bergstrom DA Uetsuki T Dac-Korytko I Sun YH Wright WE Tapscott SJ Kamps MP A conserved motif N-terminal to the DNA-binding domains of myogenic bHLH transcription factors mediates cooperative DNA binding with pbx-Meis1/Prep1 Nucleic Acids Res 1999 27 3752 3761 10471746 10.1093/nar/27.18.3752 PMC148632

[B7] Morgan R In der Rieden P Hooiveld MH Durston AJ Identifying *HOX* paralog groups by the PBX-binding region Trends Genet 2000 16 66 67 10652532 10.1016/s0168-9525(99)01881-8

[B8] McGinnis W Krumlauf R Homeobox genes and axial patterning Cell 1992 68 283 302 1346368 10.1016/0092-8674(92)90471-n

[B9] Miller GJ Miller HL van Bokhoven A Lambert JR Werahera PN Schirripa O Lucia MS Nordeen SK Aberrant *HOX*C expression accompanies the malignant phenotype in human prostate Cancer Res 2003 63 5879 5888 14522913

[B10] Norris JD Chang CY Wittmann BM Kunder RS Cui H Fan D Joseph JD McDonnell DP The homeodomain protein *HOX*B13 regulates the cellular response to androgens Mol Cell 2009 36 405 416 19917249 10.1016/j.molcel.2009.10.020 PMC2788777

[B11] Waltregny D Alami Y Clausse N de Leval J Castronovo V Overexpression of the homeobox gene *HOX*C8 in human prostate cancer correlates with loss of tumor differentiation Prostate 2002 50 162 169 11813208 10.1002/pros.10045

[B12] Eklund EA The role of *HOX* genes in malignant myeloid disease Curr Opin Hematol 2007 14 85 89 17255784 10.1097/MOH.0b013e32801684b6

[B13] Huang L Pu Y Hepps D Danielpour D Prins GS Posterior *HOX* gene expression and differential androgen regulation in the developing and adult rat prostate lobes Endocrinology 2007 148 1235 1245 17138648 10.1210/en.2006-1250 PMC2276874

[B14] Ando H Natsume A Senga T Watanabe R Ito I Ohno M Iwami K Ohka F Motomura K Kinjo S Ito M Saito K Peptide-based inhibition of the *HOX*A9/PBX interaction retards the growth of human meningioma Cancer Chemother Pharmacol 2014 73 53 60 24141373 10.1007/s00280-013-2316-5

[B15] Daniels TR Neacato II Rodriguez JA Pandha HS Morgan R Penichet ML Disruption of *HOX* activity leads to cell death that can be enhanced by the interference of iron uptake in malignant B cells Leukemia 2010 24 1555 1565 20574452 10.1038/leu.2010.142 PMC3743965

[B16] Errico MC Felicetti F Bottero L Mattia G Boe A Felli N Petrini M Bellenghi M Pandha HS Calvaruso M Tripodo C Colombo MP The abrogation of the *HOX*B7/PBX2 complex induces apoptosis in melanoma through the miR-221&222-c-FOS pathway Int J Cancer 2013 133 879 892 23400877 10.1002/ijc.28097 PMC3812682

[B17] Li Z Zhang Z Li Y Arnovitz S Chen P Huang H Jiang X Hong GM Kunjamma RB Ren H He C Wang CZ PBX3 is an important cofactor of *HOX*A9 in leukemogenesis Blood 2013 121 1422 1431 23264595 10.1182/blood-2012-07-442004 PMC3578957

[B18] Morgan R Boxall A Harrington KJ Simpson GR Gillett C Michael A Pandha HS Targeting the *HOX*/PBX dimer in breast cancer Breast Cancer Res Treat 2012 136 389 398 23053648 10.1007/s10549-012-2259-2

[B19] Morgan R Pirard PM Shears L Sohal J Pettengell R Pandha HS Antagonism of *HOX*/PBX dimer formation blocks the *in vivo* proliferation of melanoma Cancer Res 2007 67 5806 5813 17575148 10.1158/0008-5472.CAN-06-4231

[B20] Morgan R Plowright L Harrington KJ Michael A Pandha HS Targeting *HOX* and PBX transcription factors in ovarian cancer BMC Cancer 2010 10 89 20219106 10.1186/1471-2407-10-89 PMC2846885

[B21] Plowright L Harrington KJ Pandha HS Morgan R *HOX* transcription factors are potential therapeutic targets in non-small-cell lung cancer (targeting *HOX* genes in lung cancer) Br J Cancer 2009 100 470 475 19156136 10.1038/sj.bjc.6604857 PMC2658540

[B22] Shears L Plowright L Harrington K Pandha HS Morgan R Disrupting the interaction between *HOX* and PBX causes necrotic and apoptotic cell death in the renal cancer lines CaKi-2 and 769-P J Urol 2008 180 2196 2201 18804814 10.1016/j.juro.2008.07.018

[B23] Stone KR Mickey DD Wunderli H Mickey GH Paulson DF Isolation of a human prostate carcinoma cell line (DU 145) Int J Cancer 1978 21 274 281 631930 10.1002/ijc.2910210305

[B24] Kaighn ME Narayan KS Ohnuki Y Lechner JF Jones LW Establishment and characterization of a human prostatic carcinoma cell line (PC-3) Invest Urol 1979 17 16 23 447482

[B25] Horoszewicz JS Leong SS Kawinski E Karr JP Rosenthal H Chu TM Mirand EA Murphy GP LNCaP model of human prostatic carcinoma Cancer Res 1983 43 1809 1818 6831420

[B26] Webber MM Bello D Quader S Immortalized and tumorigenic adult human prostatic epithelial cell lines: characteristics and applications part 2. Tumorigenic cell lines Prostate 1997 30 58 64 9018337 10.1002/(sici)1097-0045(19970101)30:1<58::aid-pros9>3.0.co;2-h

[B27] Workman P Balmain A Hickman JA McNally NJ Rohas AM Mitchison NA Pierrepoint CG Raymond R Rowlatt C Stephens TC UKCCCR guidelines for the welfare of animals in experimental neoplasia Lab Anim 1988 22 195 201 3172698 10.1258/002367788780746467

[B28] Sauvageau G Lansdorp PM Eaves CJ Hogge DE Dragowska WH Reid DS Largman C Lawrence HJ Humphries RK Differential expression of homeobox genes in functionally distinct CD34+ subpopulations of human bone marrow cells Proc Natl Acad Sci U S A 1994 91 12223 12227 7527557 10.1073/pnas.91.25.12223 PMC45409

[B29] Vider BZ Zimber A Hirsch D Estlein D Chastre E Prevot S Gespach C Yaniv A Gazit A Human colorectal carcinogenesis is associated with deregulation of homeobox gene expression Biochem Biophys Res Commun 1997 232 742 748 9126347 10.1006/bbrc.1997.6364

[B30] Kalra N Kumar V c-Fos is a mediator of the c-myc-induced apoptotic signaling in serum-deprived hepatoma cells via the p38 mitogen-activated protein kinase pathway J Biol Chem 2004 279 25313 25319 15078869 10.1074/jbc.M400932200

[B31] Mikula M Gotzmann J Fischer AN Wolschek MF Thallinger C Schulte-Hermann R Beug H Mikulits W The proto-oncoprotein c-Fos negatively regulates hepatocellular tumorigenesis Oncogene 2003 22 6725 6738 14555986 10.1038/sj.onc.1206781

[B32] Patel P Young JG Mautner V Ashdown D Bonney S Pineda RG Collins SI Searle PF Hull D Peers E Chester J Wallace DM A phase I/II clinical trial in localized prostate cancer of an adenovirus expressing nitroreductase with CB1954 [correction of CB1984] Mol Ther 2009 17 1292 1299 19367257 10.1038/mt.2009.80 PMC2835198

[B33] Sonpavde G Thompson TC Jain RK Ayala GE Kurosaka S Edamura K Tabata K Ren C Goltsov AA Mims MP Hayes TG Ittmann MM GLIPR1 tumor suppressor gene expressed by adenoviral vector as neoadjuvant intraprostatic injection for localized intermediate or high-risk prostate cancer preceding radical prostatectomy Clin Cancer Res 2011 17 7174 7182 21933889 10.1158/1078-0432.CCR-11-1899 PMC3865786

[B34] Thirukkumaran CM Nodwell MJ Hirasawa K Shi ZQ Diaz R Luider J Johnston RN Forsyth PA Magliocco AM Lee P Nishikawa S Donnelly B Oncolytic viral therapy for prostate cancer: efficacy of reovirus as a biological therapeutic Cancer Res 2010 70 2435 2444 20215509 10.1158/0008-5472.CAN-09-2408

[B35] Chou R Dana T Bougatsos C Fu R Blazina I Gleitsmann K Rugge JB Treatments for Localized Prostate Cancer: Systematic Review to Update the 2002 U.S. Preventive Services Task Force Recommendationed 2011 Rockville (MD): Agency For Healthcare Research and Quality 22171386

